# Comparative Efficacy and Safety Profiles of High-power, Short-duration and Low-power, Long-duration Radiofrequency Ablation in Atrial Fibrillation: A Systematic Review and Meta-analysis

**DOI:** 10.19102/icrm.2023.14072

**Published:** 2023-07-15

**Authors:** Satesh Kumar, Mahima Khatri, Sumeet Kumar, F.N.U. Partab, F.N.U. Manoj Kumar, F.N.U. Neha, F.N.U. Suman, Lajpat Rai, F.N.U. Sangam, Simran Kumari, Hamza Islam, Rabia Islam, Tirath Patel

**Affiliations:** ^1^Department of Medicine, Shaheed Mohtarma Benazir Bhutto Medical College, Karachi, Pakistan; ^2^Department of Medicine, Dow University of Health Sciences, Karachi, Pakistan; ^3^Department of Medicine, Chandka Medical College, SMBBMU Larkana, Larkana, Pakistan; ^4^Department of Medicine, Jinnah Sindh Medical University (JSMU), Karachi, Pakistan; ^5^Department of Medicine, Ghulam Muhammad Mahar Medical College, Sukkur, Pakistan; ^6^Department of Medicine, Liaquat University of Medical and Health Science, Jamshoro, Pakistan; ^7^Department of Medicine, Punjab Medical College, Faisalabad, Pakistan; ^8^Department of Medicine, American University of Antigua, Osbourn, Antigua and Barbuda

**Keywords:** Atrial fibrillation, high power short duration, low power long duration, meta-analysis, radiofrequency ablation

## Abstract

High-power, short-duration (HPSD) radiofrequency (RF) ablation is expected to be more effective and safer than low-power, long-duration (LPLD) RF ablation in treating atrial fibrillation (AF). Given the limited data available, the findings are controversial. This meta-analysis evaluated whether the clinical effects of HPSD outweigh those of LPLD. A systematic search of PubMed, Embase, and Google Scholar databases identified studies comparing HPSD to LPLD ablation. All the analyses used the random-effects model. This analysis included 21 studies with a total of 4,169 patients. Pooled analyses revealed that HPSD was associated with a lower recurrence of atrial tachyarrhythmias (ATAs) at 1 year (relative risk [RR], 0.62; 95% confidence interval [CI], 0.50–0.78; *P* = .00001; *I*^2^ = 0%). Furthermore, the HPSD approach reduced the risk of AF recurrence (RR, 0.64; 95% CI, 0.40–1.01; *P* = .06; *I*^2^ = 86%). The HPSD approach was associated with a lower risk of esophageal thermal injury (ETI) (RR, 0.78; 95% CI, 0.58–1.04; *P* = .09; *I*^2^ = 73%). The HPSD strategy increased first-pass pulmonary vein (PV) isolation (PVI) and decreased acute PV reconnection (PVR), both of which were predominantly manifested in bilateral and left PVs. HPSD facilitated a reduction in procedural time, number of lesions created during PVI, and fluoroscopy time. The HPSD method reduces ETI, PVR, and recurrent AF. The HPSD approach also reduced the procedural time, number of lesions created during PVI, fluoroscopy time, and post-ablation AF relapse in 1 year, improving patient outcomes and safety.

## Introduction

Catheter ablation (CA) has become the most widely accepted ablation technique for atrial fibrillation (AF) in cardiology.^1^ It has been shown to reduce symptoms and regulate the heart rate and rhythm effectively. Although CA is a relatively secure process with limited periprocedural adverse effects, several relevant factors must be considered when recognizing patients with comparatively lower success rates or a relatively high number of postoperative complications, including concurrent heart problems, obesity, sleep problems, left atrial (LA) size, age at diagnosis, and vulnerability.^1,2^ To evaluate the incidence of symptoms associated with AF and the use of CA to treat it from 2016–2018, a technique was employed that relied on the 2014 guidelines issued by the American Heart Association and the American College of Cardiology as a standard of reference. Class I indications for this procedure included symptomatic paroxysmal AF (PAF) unresponsive to ≥1 potential therapeutic drug, class IIA indications included persistent AF (PeAF) unresponsive to ≥1 anti-arrhythmic drug (AAD), and class IIB indications included PeAF.^3^ In the 2019-centered revision, CA for symptomatic AF and cardiac failure with a lower ejection fraction was incorporated into class IIB to lessen heart failure hospital stays and mortality. This modification does not impact the category of guidelines for any patient populations included in the current analysis; however, cases meeting this implication may not be adequately portrayed in the sample population.^3^

The myocardial thickness varies in different parts of the LA, making it a frame of considerable complexity. Impactful pulmonary vein (PV) isolation (PVI) necessitates a comprehensive insight into the adjacent tissue that might be harmed by extreme radiofrequency (RF) power application to be achieved safely.^4^ For PVI, it is recommended to perform RF ablation over a large area.^5^ RF energy is conveyed as low-power, long-duration (LPLD) lesions steered by factors such as the force–time integral; however, the ideal ablation specifications to accomplish resilient, long-term PVI in a relatively secure manner remain to be ascertained.^4,5^ HPSD lesions are gaining popularity due to the resistive heating and scarring of extracardiac frameworks; this technique may minimize procedure and fluoroscopy durations, as well as the risk of adverse effects.^6^ Conductive heating dominates the LPLD RF ablation. Compared to conventional ablation, HPSD ablation produces a broader domain of specific resistive heating of parenchyma with a more rapid temperature deterioration. This skyrockets resistance to conductive tissue heating and irreversibly damages the impedance endocardial zone. Many centers use catheters to isolate PVs and create extra PV lesion configurations. Each site receives 20–40 W for 20–40 s at a contact force (CF) of 10–20 g. HPSD ablation is appealing due to lengthy procedural times and rising PV reconnection (PVR) rates. HPSD ablation is defined arbitrarily as 40–90 W for <15 s per lesion.^7^

In the past, several randomized controlled trials (RCTs) have been carried out to compare the efficacy and safety of the HPSD approach with LPLD. However, the majority of studies produced transient and inconsistent results due to short-term follow-up results and the need for more recently available studies (RCTs and observational studies). In this meta-analysis, we report findings after analyzing the available literature on this topic. Based on an extensive literature search, we conclude that this is the most recent updated meta-analysis assessing the safety and efficacy profile of conventional LPLD with the newly adopted HPSD technique for PVI.

## Methodology

This meta-analysis adheres to the Preferred Reporting Items for Systematic Review and Meta-analysis (PRISMA) guidelines.^8,9^

### Data sources and search strategy

The databases of PubMed, Ovid, the Cochrane Library, and Elsevier’s ScienceDirect were thoroughly electronically searched without language restrictions for clinical studies (updated in January 2023). To retrieve literature, straightforward keyword and medical subject heading (MeSH) term combinations (such as “high power short duration,” “atrial fibrillation,” “catheter ablation,” “radiofrequency ablation,” etc.) were used. Information about the search methodology is provided in **Supplementary Table 1**. The PICO (population, intervention, comparison, and outcome) approach was modified. The population of interest included patients with AF (PAF and PeAF). PVI (additional lesions) was performed in the treatment of AF using RF CA. The ablation energy was compared: HPSD versus LPLD. Three researchers (M.K., Sa. K., and Su. K.) independently reviewed the titles and abstracts of potentially eligible studies.

### Inclusion and exclusion criteria

Eligible studies included (1) RCTs, cohort studies, case–control studies, and cross-sectional studies; (2) studies in which an HPSD approach (>40 W) was used in the isolation of PVs; (3) studies exhibiting homogeneity of baseline data across compared groups; and (4) studies with a comparative setup. Separately, (1) non-clinical studies; (2) studies without controls; (3) conference abstracts, case reports, case series studies, editorials, and review articles; (4) studies with a sample size of <20 participants; (5) studies with equivocal results; and (6) studies without full-text versions available were excluded.

### Data extraction and definitions

The data from each study, including the author’s name, year of publication, country, study population, demographic data of participants, ablation procedure strategies, and clinical outcomes, were extracted into a specific data-collection form. The primary outcomes were atrial tachyarrhythmias (ATAs) and AF, atrial tachycardia/atrial flutter (AT/AFL) recurrence post-blanking (2 or 3 months post-ablation depending on the studies included), and major complications. The latter included esophageal thermal injury (ETI) and CA-related heart complications such as cardiac tamponade, among others. Secondary outcomes included first-pass pulmonary vein isolation (FPI) and acute PVR, procedural time, PVI ablation number, RF time, and fluoroscopy time.

High power was defined as >40 W, and the extracted data were separated into the high-power (HP) group and the low-power (LP) group. ATA recurrence was defined as symptomatic or asymptomatic ATAs lasting >30 s after the blanking period post-ablation. ETI was defined as the esophageal collateral thermal injury brought on by ablation. Endoscopy and/or magnetic resonance imaging late gadolinium enhancement was used to evaluate the morphology of the abnormal esophageal inner exhibition. An abnormal temperature increase in the esophageal temperature monitor’s reported value was also considered. First-pass PVI was defined as the first-pass RF-delivery PVI achievement rate. Adenosine test and/or waiting time was used to measure the rate of PV electrical reconnection following the first-pass ablation in acute PVR. Procedural time was defined as the time between the beginning of anesthesia and the removal of all sheaths. Fluoroscopy time was the total amount of time spent using a fluoroscope during the procedure.

### Quality of included studies

**Figure 1:**
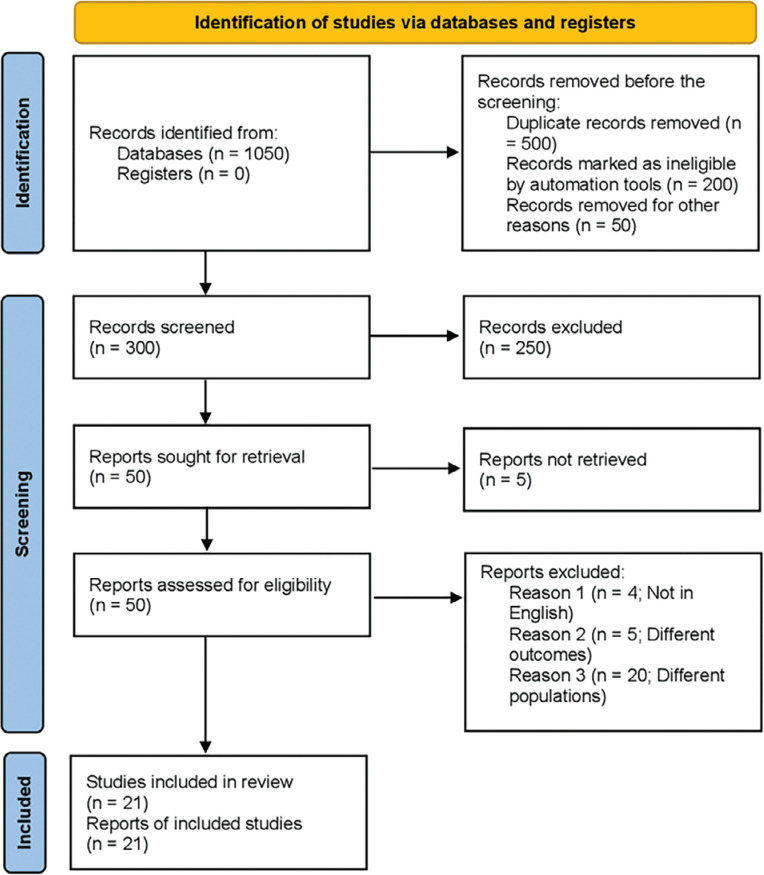
Preferred Reporting Items for Systematic Review and Meta-analysis flowchart.

A quality assessment of all the included RCTs and observational studies was performed using the Cochrane risk-of-bias tool^9^ and the Newcastle–Ottawa scale^10^ **(Supplementary Table 2 and Figure 1)**.

### Data analysis

Statistical analysis was done only for comparative studies using Review Manager version 5.4.1 (Cochrane Collaboration, London, UK). This meta-analysis presents a pooled effect of relative risks (RRs) for dichotomous outcomes and weighted mean differences for continuous outcomes calculated using the generic inverse variance with a random-effects model. All *P* values <0.05 were deemed statistically significant. The results of pooled analyses were displayed through forest plots. Funnel plots for all primary outcomes were visualized to assess publication bias. Heterogeneity was evaluated using Higgin’s *I*^2^ test, with resultant values corresponding to low (<25%), moderate (25%–75%), or high (>75%) heterogeneity.^11^ A sensitivity analysis was performed to assess the influence of the individual studies on the overall results by omitting a single study at a time when substantial heterogeneity (*I*^2^ > 75%) was present. *P* <0.05 was considered significant for all analyses.

## Results

The literature review initially yielded 1,050 articles. After removing duplicates and screening studies based on their titles and abstracts, a total of 21^12–32^ studies, including both retrospective and prospective studies, were found. Comparative studies composed the entirety of those included in this meta-analysis. The PRISMA diagram illustrates a comprehensive search strategy **(Figure 1)**. This collection of articles spans the years 2018–2022.

### Characteristics of patients

There were a total of 4,169 participants (2,285 in the HP group and 1,884 in the LP group), with a mean age ranging from 58.2 ± 10.0 to 69.0 ± 11.8 years and a follow-up duration ranging from 2–3 years. A total of 2,803 men (66.8%) and 1,366 women (32.7%) were included, and 2,258 patients (54.16%) with PAF and 1,911 patients (45.8%) with PeAF were enrolled, respectively. Age, sex, CHA_2_DS_2_-VASc score, LA diameter, hypertension, diabetes mellitus, and other relative characteristics were comparable between the 2 groups. CF-sensing irrigated catheters (ThermoCool SmartTouch^®^ NaviStar™ or ThermoCool SurroundFlow NaviStar™ [Biosense Webster, Diamond Bar, CA, USA] or TactiCath™ Quartz [Abbott, Chicago, IL, USA]) were used in all 21 studies. In 1 study, irrigated catheters that did not have CF sensors (ThermoCool SF [Biosense Webster] and FlexAbility™ [Abbott])) were used^19^ (LP group). The drag lesion technique and continuous point-by-point focal RF technique were used in 13 and 4 studies, respectively. Another 2 studies^14,15^ did not mention the ablation strategy, while 2 other studies^17,19^ used both techniques. Every patient was treated with the circumferential pulmonary vein isolation (CPVI) procedure protocol. In 9 studies, additional linear ablations (box isolation, synaptic vesicle isolation, cryo-impulse isolation, mitral isthmus isolation, and roofline isolation) as well as matrix modification (low-voltage zone or complex fractionated atrial electrogram ablation) or not were carried out. Six and 8 studies, respectively, mentioned the AADs being discontinued for 5 half-lives prior to the procedure and being maintained post-ablation. **Tables 1 and 2** detail the baseline characteristics of the patients.

### Quality assessment and publication bias

**Supplementary Figure 1:**
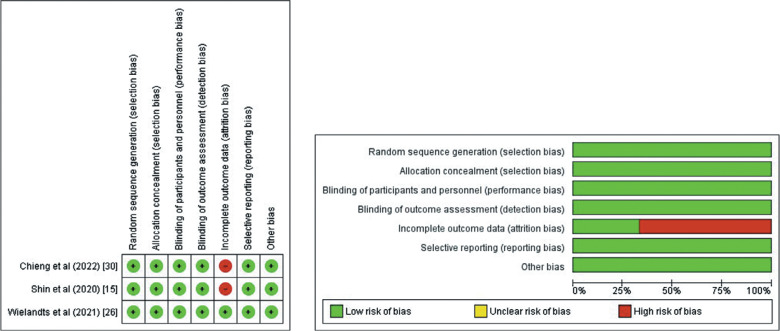
Cochrane risk-of-bias tool for included randomized controlled trials.

**Supplementary Figure 2:**
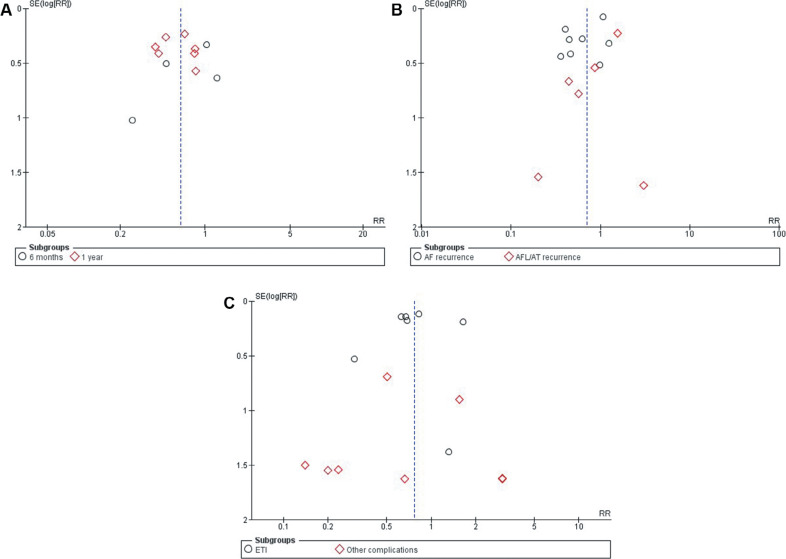
Funnel plots of primary outcomes. **A:** Recurrence of atrial tachyarrhythmias. **B:** Recurrence of atrial fibrillation and atrial tachycardia/atrial flutter. **C:** Major complications. *Abbreviations:* AF, atrial fibrillation; AFL/AT, atrial flutter/atrial tachycardia; ETI, esophageal thermal injury; RR, risk ratio; SE, standard error.

The Newcastle–Ottawa scale, a tool used to assess study quality, discovered a low likelihood of bias in observational studies **(Supplementary Table 2)**. Using the Cochrane method of assessing RCTs, we found trials of medium to high quality **(Supplementary Figure 1)**. The results were unaffected by publication bias, as demonstrated by funnel plots **(Supplementary Figure 2)**.

### Primary efficacy outcomes

**Figure 2:**
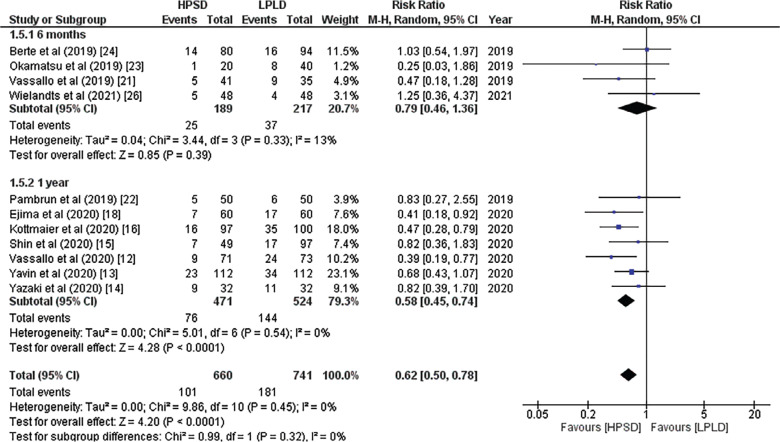
Forest plot of recurrence of atrial tachyarrhythmias. *Abbreviations:* CI, confidence interval; HPSD, high-power short-duration; LPLD, low-power long-duration; M-H, Mantel–Haenszel; RR, relative risk.

**Figure 3:**
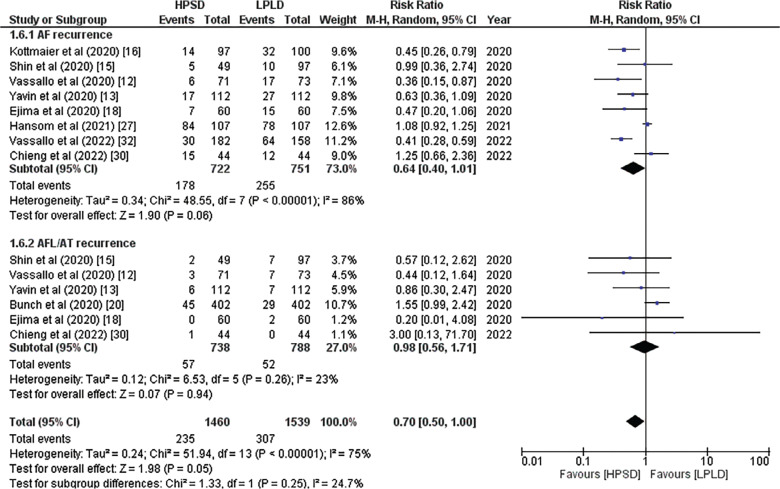
Forest plot showing subgroup analysis of recurrence of atrial fibrillation and atrial tachycardia/atrial flutter. *Abbreviations:* CI, confidence interval; HPSD, high-power short-duration; LPLD, low-power long-duration; M-H, Mantel–Haenszel; RR, relative risk.

In a pooled analysis, seven studies found that the HP approach was associated with a lower recurrence of ATAs at 1 year of follow-up (RR, 0.62; 95% CI, 0.50–0.78; *P* = .00001; *I*^2^ = 0%; **Figure 2**). In contrast, there was no statistically significant difference in the rate of ATA relapse after 6 months of follow-up (RR, 0.79; 95% CI, 0.46–1.36; *P* = .39; *I*^2^ = 13%; **Figure 2**). Eight studies indicated a lower AF recurrence in the HP group in the subgroup analysis (RR, 0.64; 95% CI, 0.40–1.01; *P* = .06; *I*^2^ = 86%; **Figure 3**), while 6 studies indicated similar AT/AFL recurrence rates in both groups (RR, 0.98; 95% CI, 0.56–1.71; *P* = .94; *I*^2^ = 23%; **Figure 3**). Due to the high heterogeneity in AF recurrence, a “leave-one-out” analysis was performed, which revealed that excluding the study by Hansom et al.^27^ reduced the in-study heterogeneity and made the results statistically significant (RR, 0.57; 95% CI, 0.40–0.80; *P* = .001; *I*^2^ = 50%).

### Primary safety outcomes

**Figure 4:**
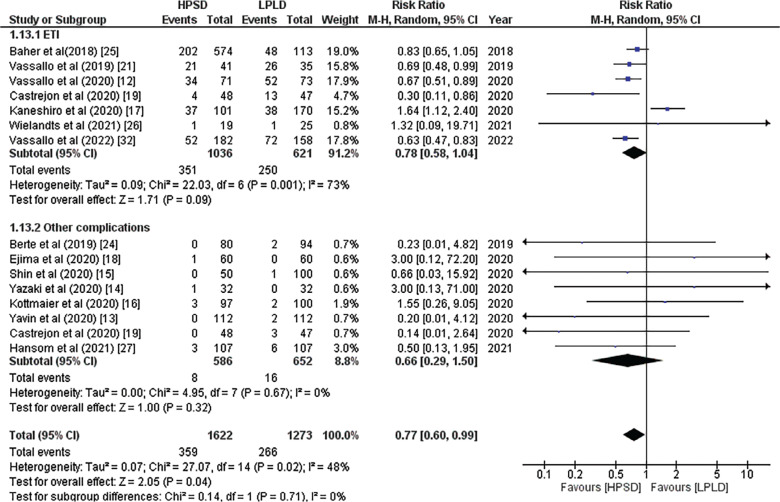
Forest plot of major complications showing subgroup analysis of esophageal thermal injury and other complications. *Abbreviations:* CI, confidence interval; HPSD, high-power short-duration; LPLD, low-power long-duration; M-H, Mantel–Haenszel; RR, relative risk.

The pooled analysis of 7 studies that included ETI found that the HP approach was linked to a lower risk of ETI (RR, 0.78; 95% CI, 0.58–1.04; *P* = .09; *I*^2^ = 73%; **Figure 4**). When included studies were gradually eliminated because of high heterogeneity, it became clear that leaving out the study by Kaneshiro et al.^17^ reduced heterogeneity and improved the statistical significance of the findings (RR, 0.70; 95% CI, 0.61–0.81; *P* = .00001; *I*^2^ = 5%). The HP approach was linked to a lower risk of other complications, such as pericardial tamponade, atrio-esophageal fistula, stroke, and phrenic nerve injury, according to 8 studies that reported these issues (RR, 0.66; 95% CI, 0.29–1.50; *P* = .32; *I*^2^ = 0%; **Figure 4**).

### Secondary outcomes

This study demonstrated that the HP group had a higher overall rate of FPI. The FPI rate was significantly higher for bilateral PVs. The leave-one-out analysis, prompted by the high in-study heterogeneity, showed that no particular study impacted the outcomes. The HP approach was associated with a significantly reduced rate of acute PVR, primarily seen in the left and bilateral PV subgroups. The HPSD strategy significantly reduced the procedural time, and sensitivity analysis was performed to determine the cause of high heterogeneity, which revealed that no study affected the results. The HP group’s overall fluoroscopy time was significantly lower, primarily when PVI was used alone as opposed to when other strategies were used along with PVI. The significantly reduced ablation time, ablation number for PVI, ablation index, and total RF time are all results of HPSD treatment.

Nevertheless, the groups showed no discernible difference in impedance reduction. A leave-one-out sensitivity analysis was conducted for each outcome due to the high heterogeneity in these results, and the results showed that no single study had an impact on these findings. **Table 3** provides numerical summaries of the aforementioned results.

## Discussion

The prevalence of AF is rising, notably so in developed countries, as the population ages and the strain of cardiovascular disease rises.^33^ AF is commonly induced by ectopy in the PVs; hence, CPVI is included in most AF ablation procedures.^33^ Ablation heats and necrotizes tissue at the catheter tip by passing an RF energy current through the parenchyma. The majority of the ablation lesion is formed by heat transfer from resistive heating deeper in the tissue. RF power-generation substantially augments the depth of resistive and conductive heating.^33,34^ HPSD ablation definitions currently range from 50–90 W for durations ranging from 2–20 s. For apparent patient safety purposes, the bulk of human experimentations to date have employed a peak energy of 50 W. The HPSD technique for AF CA is more proficient than the LPLD approach due to the reduced procedural, fluoroscopy, and ablation times. Compared to LPLD, the HPSD strategy lowers the risk of ETI and leads to higher rates of FPI. Furthermore, after a single RF CA procedure, the HPSD ablation technique effectively reduces the likelihood of PVR and recurrent AF.^33,35^ The potential for the HPSD technique to increase FPI and reduce PV restoration and recurrent AF has been due to the HPSD method’s ability to generate a lesion with a broader area, greater uniformity, and greater consistency.^35^

### Main findings

In this systematic review and meta-analysis, we included 21 studies comprising 4,169 participants to compare the efficacy and safety profile of HPSD with the LPLD approach. Our findings suggested that the HPSD ablation technique is associated with a drop in the risk of recurrent ATAs and a drop in AF recurrence at 1 year of follow-up. However, the 6-month follow-up showed no statistically significant variation in ATA relapse rates. In addition, the AT/AFL recurrence rate did not differ between the 2 groups, which is a significant departure from the findings of previous meta-analyses.^5^

From a safety standpoint, our meta-analysis demonstrated that implementing RF ablation for AF using the HPSD strategy may decrease the likelihood of ETI. In percentage terms, however, this result was more significant than previous meta-analyses.^5,35^ The HP approach was also associated with a reduced risk of complications such as pericardial tamponade, atrio-esophageal fistula, stroke, and phrenic nerve injury. The HPSD approach increased FPI and decreased the acute PVR rate. In contrast with previous research, however, these findings did not demonstrate a reduction of more than half. Therefore, these variations in outcomes call into question previous studies’ methodologies. The HPSD technique was strongly correlated with superior procedural characteristics, including reduced procedural time, ablation time for PVI, and fluoroscopy duration, thereby enhancing the efficacy of AF ablation. The results manifested a variety of heterogeneities, which were most evident in the retrospective cohort studies. Numerous studies contributed significantly to the high heterogeneity, necessitating a leave-one-out sensitivity analysis for each outcome.^17,27^ This was due to the operator’s freedom to choose the size of the isolation circle surrounding the PVs, the power setting, the CF, the ablation endpoints, and the inclusion of ablation. The LA’s anatomy also varied by ethnicity. Due to measurement mistakes and lost data, earlier cohort studies exaggerated these discrepancies. However, primary and secondary outcome data were more evenly distributed across prospective trials.

### Lesion variations and complications

Human research has evaluated more powerful and shorter-duration ablations. Gastroesophageal fistulas and pulmonary stenosis were too rare to study in those studies. Nilsson et al.^36^ compared 30 W for 120-s and 45 W for 20-s ablation procedures, and, in both groups, the long-term outcomes and consequences were identical. The higher-power, shorter-duration ablation group had reduced isolation times, mean fluoroscopy times, radiation dosages, and total RF application times.^36^ In previous studies, the minimum power used was 50 W for 5 s, which produced a mean depth of 2.3 mm. As all lesions were transmural, the only limitation was tissue thickness. If the tissue is thicker, the lesion may also be thicker. Studies that have tried to deliver 50 W continuously have often had to reduce the power because of hyperechogenic tissue changes observed on intracardiac echocardiography, a sharp interim impedance drop, or a lack of transmural lesions in relatively thick cardiac muscle (mitral annulus and the septal side of the right superior PV). In the in vitro model, 40 W for 5 s was insufficient to produce a 2-mm cutoff point.^13,37^ In vitro, Ali-Ahmed et al.’s study indicated that a higher power setting (50 W for 5 s) produced a larger lesion with a maximum width of 7.2 versus 5.7 mm and a depth of 2.9 versus 2.1 mm within the identical RF period and irrigation stream (2 mL/min).^38^ According to Bhaskaran et al.’s study, the lesion thickness at 80 W for 5 s (6.5 mm) and 70 W for 5 s (5.9 mm) was greater than that at 40 W for 30 s (5.2 mm) when the CF was 10 g. In vitro, the depth was comparable (2.9 mm [80 W for 5 s] vs. 2.6 mm [70 W for 5 s] vs. 2.7 mm [40 W for 30 s], respectively).^37^ Additionally, catheter stability is a significant factor in lesion structure. Longer-duration power implementations may result in breached catheter contact consistency. Lesion features ascertained in ex vivo static tissue preparations do not precisely reflect the spectrum of movement observed in a beating heart, where there is greater variance among lesions and relatively small lesion dimensions, especially when compared to ablation in a fixed muscle formation.^33^ An atrio-esophageal fistula following LA ablation is extremely uncommon and frequently fatal.^33,34,37,38^

Even though this analysis produced sufficient statistical evidence, it is essential to note its limitations. First, variations in research design; intervention strategies; and patient characteristics such as body mass index, age, sample sizes, ethnic background, and trial attributes may have caused clinical heterogeneity. Second, the follow-up durations of the majority of studies were different, with some studies reporting longer durations. When evaluating such techniques, longer-term follow-up is more valuable. Finally, several studies employed different power levels (35, 45, 50, 60, or 70 W) at various weeks, which may have introduced ambiguity.

## Conclusion

Based on the results of this systematic review and meta-analysis, we conclude that the HPSD method reduces the likelihood of ETI, improves FPI, and reduces the risk of PVR and recurrent AF. The HPSD technique was strongly correlated with decreased procedural time, ablation number for PVI, fluoroscopy time, and post-ablation AF relapse at 1 year of follow-up, thereby improving clinical outcomes with enhanced safety.

## Figures and Tables

**Table 1:** Demographics and Comorbidities of Patients

Study	Age (Mean ± SD), years		Male No. (%)		LVEF (Mean ± SD)		CHA_2_DS_2_-VASc		LAD (Mean ± SD), mm		BMI (Mean ± SD), kg/m^2^		Hypertension No. (%)		DM No. (%)		Stroke/TIA No. (%)	
	HPSD	LPLD	HPSD	LPLD	HPSD	LPLD	HPSD	LPLD	HPSD	LPLD	HPSD	LPLD	HPSD	LPLD	HPSD	LPLD	HPSD	LPLD
Vassallo et al. (2021)^12^	59.7	60.7	50 (70.4)	50 (68.4)	N/A	N/A	2.57 ± 5.92	2.22 ± 5.18	N/A	N/A	N/A	N/A	52 (73.2)	53 (72.6)	20 (28.1)	14 (19.1)	10 (14)	8 (10.9)
Yavin et al. (2020)^13^	62.3 ± 5.2	64.8 ± 7.2	71 (63.3)	79 (70.5)	60.3 ± 6.1	57.8 ± 5.4	2.4 ± 1.3	2.6 ± 1.4	44.2 ± 4.7	47.1 ± 5.1	47.1 ± 5.1	27.6 ± 3.9	70 (62.5)	76 (67.8)	112 (9.8)	7 (6.2)	N/A	N/A
Yazaki et al. (2020)^14^	66 ± 11	61 ± 11	27 (84)	20 (63)	55 ± 7	56 ± 7	2	2	40 ± 13	41 ± 14	N/A	N/A	N/A	N/A	N/A	N/A	N/A	N/A
Shin et al. (2020)^15^	58.5 ± 7.9	58.7 ± 11.1	39 (78)	33 (66)	55.7 ± 11.4	58.9 ± 8.3	1.6 ± 1.5	1.7 ± 1.6	39.9 ± 4.6	40.7 ± 6.5	23.8 ± 2.8	24.6 ± 2.7	24 (48)	22 (44)	8 (16)	8 (16)	7 (14)	6 (12)
Kottmaier et al. (2020)^16^	60.8 ± 13.9	60.8 ± 10.5	57 (58)	60 (60)	57 ± 5	55 ± 9	1.95	1.64	N/A	N/A	27.9 ± 4.0	28.0 ± 4.5	56 (57.7)	58 (58)	N/A	N/A	6 (6.1)	7 (7)
Kaneshiro et al. (2020)^17^	63 ± 10	61 ± 10	77 (76.2)	138 (81.2)	N/A	N/A	N/A	N/A	40.8 ± 6.3	38.8 ± 6.5	24.9 ± 4.0	24.5 ± 3.7	N/A	N/A	N/A	N/A	N/A	N/A
Ejima et al. (2020)^18^	63 ± 11.3	66.7 ± 8.9	44 (73)	42 (70)	57.7 ± 3.9	57.4 ± 6.3	1.8 ± 1.4	2.2 ± 1.4	34.3 ± 10.3	36.1 ± 8.7	23.9 ± 2.8	23.8 ± 3.2	29 (48)	30 (50)	10 (17)	12 (20)	6 (10)	7 (12)
Castrejón-Castrejón et al. (2020)^19^	61 ± 10	60 ± 10	32 (67)	28 (60)	57 ± 9	56 ± 11	N/A	N/A	N/M HP, 40 (85%); M/S HP, 7 (15%)	N/M LP, 36 (82%); M/S LP, 8 (18%)	28 ± 4	29 ± 5	N/A	N/A	N/A	N/A	N/A	N/A
Bunch et al. (2020)^20^	67.1 ± 10.5	66.4 ± 12.2	253 (62.9)	262 (65.2)	54.6 ± 12.1	54.7 ± 12.8	N/A	N/A	N/A	N/A	30.8 ± 7.0	30.5 ± 6.8	358 (89.1)	348 (86.6)	126 (31.3)	121 (30.1)	47 (11.6)	51 (12.6)
Vassallo et al. (2019)^21^	64 ± 10	61 ± 12	34 (83)	22 (64.7)	N/A	N/A	2 ± 1.6	2 ± 1.97	43.3 ± 6.3	41.9 ± 6.1	27	28	33 (80)	26 (64.3)	18 (43.9)	8 (22.8)	3 (7.3)	3 (8.6)
Pambrun et al. (2019)^22^	65 ± 8.2	62.5 ± 10.6	35 (70)	30 (60)	61.7 ± 5.6	61.1 ± 4.4	N/A	N/A	N/A	N/A	N/A	N/A	14 (28)	12 (24)	3 (6)	3 (6)	3 (6)	3 (6)
Okamatsu et al. (2019)^23^	65 ± 10	68 ± 8	13 (65)	15 (75)	65 ± 8.1	64 ± 5.1	2 ± 1.48	2 ± 0.74	40 ± 6	39 ± 6	24 ± 2.2	24 ± 5.1	10 (50)	10 (50)	5 (25)	1 (5)	0 (0)	0 (0)
Berte et al. (2019)^24^	62 ± 9	63 ± 9	50 (62)	67 (71)	58 ± 8	59 ± 11	N/A	N/A	N/A	N/A	N/A	N/A	N/A	N/A	N/A	N/A	N/A	N/A
Baher et al. (2018)^25^	69 ± 11.8	68.3 ± 11.6	385 (67.1)	67 (59.3)	N/A	N/A	2.9 ± 1.7	2.5 ± 1.6	N/A	N/A	N/A	N/A	369 (64.2)	68 (60.1)	112 (19.5)	18 (18.5)	81 (14.1)	7 (6.2)
Wielandts et al. (2021)^26^	64 ± 11	61 ± 11	32 (67)	33 (69)	N/A	N/A	1 ± 2.22	1 ± 2.22	39 ± 7	40 ± 7	26.4 ± 4.2	26.8 ± 4.0	N/A	N/A	N/A	N/A	N/A	N/A
Hansom et al. (2021)^27^	62 ± 9	62 ± 9	69 (65)	81 (76)	N/A	N/A	1.9	2	41 ± 0.7	41 ± 0.6	N/A	N/A	44 (41)	47 (44)	9 (8)	13 (12)	4 (4)	9 (8)
Sousa et al. (2023)^28^	61 ± 10.3	62 ± 14.8	44 (55)	53 (66)	60 ± 9.2	60 ± 3.7	2 ± 1.48	1 ± 0.74	40.9 ± 5.9	40.3 ± 4.7	27.3 ± 3.5	27.9 ± 4	47 (59)	53 (66)	7 (9)	11 (14)	8 (10)	3 (4)
Salló et al. (2022)^29^	58 ± 8.14	60 ± 6.48	27 (54)	32 (60)	55 ± 5.9	60 ± 5.9	2 ± 1.48	2 ± 1.48	43 ± 5.9	44 ± 6.6	N/A	N/A	37 (74)	33 (62)	7 (14)	8 (15)	0 (0)	8 (15)
Chieng et al. (2023)^30^	62.9 ± 8.2	59.7 ± 10	31 (77.2)	30 (68.1)	55.4 ± 13	54.6 ± 11.4	2 ± 2	1.5 ± 1.5	N/A	N/A	29.2 ± 5.6	29.2 ± 4.8	25 (56.8)	14 (31.8)	3 (6.8)	2 (4.5)	1 (2.3)	2 (4.5)
Cheng et al. (2022)^31^	58.9 ± 11.9	63.1 ± 9.9	27 (75)	20 (55.5)	63 ± 4.4	61.5 ± 4.07	1.69 ± 1.4	2.31 ± 1.9	38.1 ± 6.4	38.0 ± 6.4	24.11 ± 2.9	25.3 ± 3.2	20 (55.6)	18 (50)	6 (16.7)	7 (19.4)	2 (5.6)	4 (11.1)
Vassallo et al. (2022)^32^	58.4 ± 14	52.9 ± 10.5	131 (71.8)	113 (71.5)	61 ± 9.2	63.2 ± 7.4	2.4 ± 5.92	2 ± 5.18	42.9 ± 5.1	40.6 ± 4.25	N/A	N/A	126 (69.2)	105 (66.4)	34 (18.6)	32 (20.2)	13 (7.1)	11 (6.9)

*Abbreviations:* BMI, body mass index; CTI, cavo-tricuspid isthmus; DM, diabetes mellitus; FAT, focal atrial tachycardia; HF, heart failure; LAD, left atrial diameter; LAVI, left atrial volume index; LVA, low-voltage ablation; LVEF, left ventricular ejection fraction; M/S, moderate/severe; MIL, mitral isthmus line; N/M, normal/mild; RL, roofline; SD, standard deviation; SVC, superior vena cava; TIA, transient ischemic attack. Values are expressed as mean ± SD and no. (%) according to the original text.

**Table 2:** Study and Procedure Characteristics

Study	Study Design	Total No. of Patients	No. of Patients		Mapping Tools	Ablation Strategy	Catheter	The Proportion of PAF No. (%)		Ablation Protocol		Ablation Strategy
			HPSD	LPLD				HPSD	LPLD	HPSD	LPLD	
Vassallo et al. (2021)^12^	Retrospective cohort	144	71	73	EnSite™	CPVI	CF catheter	39 (54.93)	52 (71.23)	50 W, 10–20 g for 6 s (AW)/45 W for 6 s (PW/roof/atrial flutter)	20 W (PW) 30 W (elsewhere) 30 s	Dragging
Yavin et al. (2020)^13^	Prospective cohort	224	112	112	CARTO^®^ 3	CPVI + (RL/MIL/CTI)	CF catheter	76 (67.8)	67 (59.8)	45–50 W for 8 s (PW) or 15 s (ridge/septal/mitral annulus aspect)	20 W 20 s (PW) or 30–40 W 30 s (ridge/septal/mitral annulus aspect)	Point-by-point
Yazaki et al. (2020)^14^	Prospective cohort	64	32	32	CARTO^®^ 3	CPVI + linear ablation + focal AT ablation	CF catheter	N/A	N/A	N/A	N/A	N/A
Shin et al. (2020)^15^	RCT	100	50	50	CARTO^®^ 3	CPVI + (MAL/CTI/box)	CF catheter	25 (50)	24 (48)	50 W, <20 g for 10 s	30 W <20 g 40 s	N/A
Kottmaier et al. (2020)^16^	Prospective cohort	197	97	100	EnSite™	CPVI	CF catheter	97 (100)	100 (100)	70 W for 7 s (AW) or 5 s (PW)	30–40 W 20–40 s	Point-by-point
Kaneshiro et al. (2020)^17^	Prospective cohort	271	101	170	CARTO^®^ 3	CPVI + (box/LVA)	CF catheter	67 (66)	134 (79)	45–50 W, 10–15 g until AI 400	20–30 W	Point-by-point
Ejima et al. (2020)^18^	Prospective cohort	120	60	60	CARTO^®^ 3	CPVI + (SVC/CTI/FA) CPVI	CF catheter	60 (100)	60 (100)	50 W, 5–20 g for 3–5 s	25–40 W 10–20 g 5–10 s	Point-by-point
Castrejón-Castrejón et al. (2020)^19^	Prospective cohort	95	48	47	CARTO^®^ 3	N/A	HP: CF catheter LP: non-CF catheter	31 (65)	30 (64)	(1) 18 patients: 50 W for 30 s or (2) 30 patients: 60 W for 7 s; lesion index 5 or AI 350 (PW) 450 (elsewhere)	LP: 30 W 30 s	Point-by-point (PW) Dragging (elsewhere)
Bunch et al. (2020)^20^	Retrospective cohort	804	402	402	CARTO^®^ 3	CPVI+	CF catheter	190 (47)	202 (50)	50 W, 5–20 g for 2–3 s	30 W 5–20 g 3–10 s	Point-by-point
Vassallo et al. (2019)^21^	Retrospective cohort	76	41	35	EnSite™	CPVI	CF catheter	28 (68.3)	27 (77)	50 W, 10–20 g for 6–8 s (AW)/45 W, 8–15 g for 6–8 s (PW/roof)	30 W 10–30 g 30 s	Dragging
Pambrun et al. (2019)^22^	Prospective cohort	100	50	50	CARTO^®^ 3	CPVI	CF catheter	50 (100)	50 (100)	40–50 W, 10–20 g for >5 s until unipolar signal modification	5 W (PW), 30 W (elsewhere) 10–20 g >2 s until unipolar signal modification	Point-by-point
Okamatsu et al. (2019)^23^	Prospective cohort	40	20	20	CARTO^®^ 3	CPVI + (box/LVA/CFAE/SVC/CTI)	CF catheter	13 (65)	16 (80)	50 (AW), 40 (PW), or 30 W (esophagus), 10–20 g	30 W (AW), 20 W (PW), 20 W (esophagus) 10–20 g until AI 400 (AW), 360	Point-by-point
Berte et al. (2019)^24^	Prospective cohort	174	80	94	CARTO^®^ 3	CPVI	CF catheter	65 (81)	74 (79)	45 W (AW) or 35 W (PW)	35 W (AW) 25 W (PW) until AI 550 (AW) or 400 (PW/roof/inferior), 260 (on the esophagus), or 570 (PV carina)	Point-by-point
Baher et al. (2018)^25^	Retrospective cohort	687	574	113	CARTO^®^ 3/EnSite™	CPVI additional ablation (linear ablation and substrate modifications)	CF catheter	276 (46.8)	80 (70.8)	50 W, 10–20 g for 5 s	35 W 10–20 g 10–30 s	Dragging
Wielandts et al. (2021)^26^	RCT	96	48	48	CARTO^®^ 3	CPVI + CTI	CF catheter	48 (100)	48 (100)	45 W for <30 g	35 W <30 g	Point-by-point
Hansom et al. (2021)^27^	Prospective cohort	214	107	107	CARTO^®^ 3	Extended WACA, clustered lesions, encircling lesion set, additional lines, posterior box	CF catheter	67 (63)	60 (56)	50 W for 8–10 s per lesion (AWl/floor/roof), 50 W for 6–8 s per lesion (posterior)	FTI ≥ 400 g·s, power, ≤35 W for a duration of 30–40 s (anterior wall/floor/roof); FTI ≥ 300 g·s (not to exceed 400 g·s); power ≤ 25 W for a duration of 20–25 s (posterior)	Point-by-point
Sousa et al. (2023)^28^	Prospective cohort	160	80	80	CARTO^®^	Ostial circumferential ablation	CF catheter	N/A	N/A	40 W on the posterior wall, 50 W elsewhere	25 W on the posterior wall, 35 W elsewhere	Point-by-point
Salló et al. (2022)^29^	Prospective cohort	103	50	53	CARTO^®^ 3	CPVI	CF catheter	37 (74)	29 (55)	50 W for ≥3 s; maximum range, 2.5 mm; force over time, 30% with a minimum force of 3 g; radius of lesion tags, 3 mm	30 W for ≥3 s; maximum range, 2.5 mm; force over time, 30% with a minimum force of 3 g; radius of lesion tags, 3 mm	Point-by-point
Chieng et al. (2023)^30^	RCT	88	44	44	CARTO^®^ 3/EnSite™	CPVI	CF catheter	15 (34.1)	21 (47.7)	45 W	25 W	Point-by-point
Cheng et al. (2022)^31^	Retrospective cohort	72	36	36	CARTO^®^	CPVI	ST/STSF catheter	N/A	N/A	50 W for 31 s, AI 500	40 W for 19 s, AI 490	Point-by-point
Vassallo et al. (2022)^32^	Retrospective cohort	340	182	158	EnSite™	CPVI	CF catheter	N/A	N/A	45 or 50 W, contact force of 8–15 g or 10–20 g and 35 mL/min	30 or 20 W, 10–30 g for 30 s	Dragging

*Abbreviations:* AF, atrial fibrillation; AW, anterior wall; CF, contact force; CPVI, circumferential pulmonary vein isolation; CTI, cavo-tricuspid isthmus; HPSD, high power short duration; LPLD, low power long duration; MIL, mitral isthmus line; N/A, not available; PAF, paroxysmal AF; PW, posterior wall; RL, roof line; STS, SmartTouch^®^ SurroundFlow; SVC, superior vena cava; WACA, wide area circumferential ablation.

**Table 3:** Secondary Outcomes

Outcomes	Effect Sizes (RR or WMD)	95% CI	*P* Value	*I* ^2^
FPI	RR, 1.19	1.08–1.30	.0003	85%
Left PVs	RR, 1.09	0.99–1.20	.09	63%
Right PVs	RR, 1.07	0.97–1.18	.17	45%
Bilateral PVs	RR, 1.57	1.19–2.09	.002	95%
Acute PVR	RR, 0.57	0.45–0.73	<.00001	52%
Left PVR	RR, 0.57	0.43–0.76	.0001	0%
Right PVR	RR, 0.84	0.59–1.22	.36	0%
Bilateral PVR	RR, 0.52	0.37–0.75	.0004	69%
Total procedural time	WMD, −35.58	−41.16 to −25.01	<.00001	95%
Total fluoroscopy time	WMD, −3.16	−4.64 to −1.68	<.0001	96%
PVI	WMD, −3.06	−4.73 to −1.39	.0003	96%
PVI + additional ablation	WMD, −3.12	−6.63 to 0.40	.08	96%
Ablation time	WMD, −19.19	−24.83 to −13.55	<.00001	97%
Ablation number for PVI	WMD, −7.00	−9.74 to −4.25	<.00001	41%
Ablation index	WMD, −30.17	−54.86 to −5.48	.02	98%
Total RF time	WMD, −20.78	−26.46 to −15.10	<.00001	98%
Impedance decrease	WMD, 0.22	−1.42 to 1.87	.79	96%

*Abbreviations:* CI, confidence interval; FPI, first-pass pulmonary vein isolation; *I*^2^, heterogeneity; PVI, pulmonary vein isolation; PVR, pulmonary vein reconnection; PVs, pulmonary veins; RF time, radiofrequency time; RR, relative risk; WMD, weighted mean difference.

**Supplementary Table 1:** Detailed Search Strategy

Database	Search Strategy	Results
PubMed	((“high-power”[All Fields] AND (“short”[All Fields] OR “shorts”[All Fields]) AND (“duration”[All Fields] OR “durations”[All Fields])) OR (“high”[All Fields] AND (“power, psychological”[MeSH Terms] OR (“power”[All Fields] AND “psychological”[All Fields]) OR “psychological power”[All Fields] OR “power”[All Fields] OR “powered”[All Fields] OR “powers”[All Fields] OR “powering”[All Fields]) AND (“short”[All Fields] OR “shorts”[All Fields]) AND (“duration”[All Fields] OR “durations”[All Fields])) OR “HPSD”[All Fields]) AND ((“low”[All Fields] AND (“power, psychological”[MeSH Terms] OR (“power”[All Fields] AND “psychological”[All Fields]) OR “psychological power”[All Fields] OR “power”[All Fields] OR “powered”[All Fields] OR “powers”[All Fields] OR “powering”[All Fields]) AND “long”[All Fields] AND (“duration”[All Fields] OR “durations”[All Fields])) OR “LPLD”[All Fields]) AND (“radiofrequency ablation”[MeSH Terms] OR (“radiofrequency”[All Fields] AND “ablation”[All Fields]) OR “radiofrequency ablation”[All Fields] OR (“catheter ablation”[MeSH Terms] OR (“catheter”[All Fields] AND “ablation”[All Fields]) OR “catheter ablation”[All Fields]) OR (“ablate”[All Fields] OR “ablated”[All Fields] OR “ablates”[All Fields] OR “ablating”[All Fields] OR “ablation”[All Fields] OR “ablational”[All Fields] OR “ablations”[All Fields])) AND (“atrial fibrillation”[MeSH Terms] OR (“atrial”[All Fields] AND “fibrillation”[All Fields]) OR “atrial fibrillation”[All Fields] OR (“atrial fibrillation”[MeSH Terms] OR (“atrial”[All Fields] AND “fibrillation”[All Fields]) OR “atrial fibrillation”[All Fields] OR “afib”[All Fields]))	700
Google Scholar	(((((high-power short duration) OR (high power short duration)) OR (HPSD)) AND ((low power long duration) OR (LPLD))) AND (((radiofrequency ablation) OR (catheter ablation)) OR (ablation))) AND ((atrial fibrillation) OR (Afib))	200
Embase	(((((high-power short duration) OR (high power short duration)) OR (HPSD)) AND ((low power long duration) OR (LPLD))) AND (((radiofrequency ablation) OR (catheter ablation)) OR (ablation))) AND ((atrial fibrillation) OR (Afib))	150

**Supplementary Table 2:** Newcastle–Ottawa Scale to Assess Bias in Observational Studies

Study		Selection			Comparability		Outcomes		Total
	Representativeness of the Exposed Cohort	Selection of the Non-exposed Cohort	Ascertainment of Exposure	Demonstration That Outcome of Interest Was Not Present at Start of Study	Comparability of Cohorts on the Basis of the Design or Analysis	Assessment of Outcome	Was Follow-up Long Enough for Outcomes to Occur?	Adequacy of Cohort Follow-up	
Vassallo et al. (2021)^12^	*	*	*	*	**	*	*	*	*********
Yavin et al. (2020)^13^	*	*	*	*	*	*	*	*	********
Yazaki et al. (2020)^14^	*	*	-	*	*	*	*	*	*******
Kottmaier et al. (2020)^16^	*	*	*	*	**	*	*	*	*********
Kaneshiro et al. (2020)^17^	*	*	*	*	*	*	*	*	********
Ejima et al. (2020)^18^	*	-	*	*	**	*	*	*	********
Castrejón-Castrejón et al. (2020)^19^	*	*	*	*	*	*	*	*	********
Bunch et al. (2020)^20^	*	*	*	*	**	*	*	*	*********
Vassallo et al. (2019)^21^	*	*	*	*	**	*	*	*	*********
Pambrun et al. (2019)^22^	*	*	*	*	*	*	*	*	********
Okamatsu et al. (2019)^23^	*	*	*	*	**	*	*	*	********
Berte et al. (2019)^24^	*	*	-	*	*	*	*	*	*******
Baher et al. (2018)^25^	*	*	*	*	**	*	*	*	*********
Hansom et al. (2021)^27^	*	*	*	*	*	*	*	*	********
Sousa et al. (2023)^28^	*	*	*	-	**	*	*	*	********
Salló et al. (2022)^29^	*	*	-	*	*	*	*	*	*******
Cheng et al. (2022)^31^	*	*	*	*	*	*	*	*	********
Vassallo et al. (2022)^32^	*	*	*	*	**	*	*	*	*********

The Newcastle–Ottawa Scale quality instrument is scored by awarding points (asterisks) for each answer. The possible total points are 4 points for selection, 2 points for comparability, and 3 points for outcomes. Good quality: 3 or 4 stars in the selection domain AND 1 or 2 star(s) in the comparability domain AND 2 or 3 stars in the outcome/exposure domain. Fair quality: 2 stars in the selection domain AND 1 or 2 star(s) in the comparability domain AND 2 or 3 stars in outcome/exposure domain. Poor quality: 0 or 1 star(s) in selection domain OR 0 stars in comparability domain OR 0 or 1 star(s) in outcome/exposure domain.
